# A previously published propofol-remifentanil response surface model does not predict patient response well in video-assisted thoracic surgery: Erratum

**DOI:** 10.1097/MD.0000000000008382

**Published:** 2017-10-20

In the article, “A previously published propofol-remifentanil response surface model does not predict patient response well in video-assisted thoracic surgery”,^[1]^ which appeared in Volume 96, Issue 19 of *Medicine*, the authors wish to include the following acknowledgement:

This study was supported by the grants from Taipei Veterans General Hospital, Taipei, Taiwan, R.O.C. and Anesthesiology Research and Development Foundation, Taipei, Taiwan, R.O.C.
